# Next Generation Technology for Epidemic Prevention and Control: Data-Driven Contact Tracking

**DOI:** 10.1109/ACCESS.2018.2882915

**Published:** 2018-12-24

**Authors:** Hechang Chen, Bo Yang, Hongbin Pei, Jiming Liu

**Affiliations:** 1 College of Computer Science and TechnologyJilin University 12510 Changchun130012China; 2 Key Laboratory of Symbolic Computation and Knowledge Engineering, Ministry of EducationJilin University 12510 Changchun130012China; 3 Department of Computer ScienceHong Kong Baptist University 26679 Hong Kong

**Keywords:** Contact tracking, disease transmission, epidemic modeling, heterogeneous data mining

## Abstract

Contact tracking is one of the key technologies in prevention and control of infectious diseases. In the face of a sudden infectious disease outbreak, contact tracking systems can help medical professionals quickly locate and isolate infected persons and high-risk individuals, preventing further spread and a large-scale outbreak of infectious disease. Furthermore, the transmission networks of infectious diseases established using contact tracking technology can aid in the visualization of actual virus transmission paths, which enables simulations and predictions of the transmission process, assessment of the outbreak trend, and further development and deployment of more effective prevention and control strategies. Exploring effective contact tracking methods will be significant. Governments, academics, and industries have all given extensive attention to this goal. In this paper, we review the developments and challenges of current contact tracing technologies regarding individual and group contact from both static and dynamic perspectives, including static individual contact tracing, dynamic individual contact tracing, static group contact tracing, and dynamic group contact tracing. With the purpose of providing useful reference and inspiration for researchers and practitioners in related fields, directions in multi-view contact tracing, multi-scale contact tracing, and AI-based contact tracing are provided for next-generation technologies for epidemic prevention and control.

## Graphical abstract

Individual and group contact tracking from both static and dynamic perspectives, including static individual contact tracing, dynamic individual contact tracing, static group contact tracing, dynamic group contact tracing.

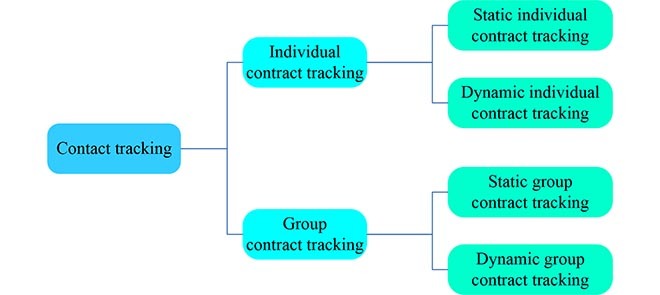

## I. Introduction

Outbreaks of infectious diseases could cause huge losses in human lives. The Spanish pandemic in 1918 led to over 20 million deaths [1]. As of 2014, approximately 3.3 billion people worldwide are at risk of malaria, and every 60 seconds one patient dies due to malaria infection [2]. The death rate of tuberculosis has exceeded AIDS, becoming the most deadly infectious disease in the world. About 80% of people in South Africa have latent tuberculosis and there were 450,000 cases of tuberculosis in 2013 alone [3].

Along with the serious threat to human lives, infectious diseases also bring huge economic losses. Statistically, malaria causes an economic loss of 12 billion US dollars every year in African countries [4]. Seasonal influenza in the US causes an annual economic burden of 87 billion US dollars [7]. The developments in vaccines and drugs have enabled us to combat infectious diseases and have greatly reduced the harm brought to human society. However, the sudden emerging infectious diseases caused by the drug resistance and the inherent variability of viruses still remains to be a serious global problem that often leaves us in an unprepared and vulnerable situation. For example, the H1N1 flu in 2009 mutated to become the H7N9 flu in 2014 and the H3N2 flu in 2015. The H3N2 virus began to spread through contact networks in Hong Kong in January 2015, and 122 lives were taken in just one month [8]. The death toll rose to 591 after three months. Thus, in the fight against various kinds of infectious diseases, relying solely on vaccine development is far from enough. A more effective “active prevention and control” method is desperately needed so as to rapidly detect and block the transmission paths of new infectious diseases, detaining the disease to a minimum spread until its eradication.

Many infectious diseases are transmitted through person-to-person “contact”. In computational epidemiology, a contact is simply defined either as a direct physical interaction (e.g. sexual contact) or proximity interaction (e.g. to people being within 1m of each other, or being in the same room) [9]. Human contact interactions constitutes a “contact network” of virus transmission. In this network, nodes represent individuals, and links represent contact relationships. The structure of the contact network significantly affects the spatiotemporal patterns of virus spread. For example, in the case of respiratory infections that spread through droplets, interactions like face-to-face communication, shaking hands, crowd gathering, and sharing vehicles enable the spread of diseases and increase the possibilities of transmission from infected to susceptible persons. Tracking the contact interactions of individuals can effectively restore the “invisible” virus transmission paths, quickly locate and isolate high-risk individuals who were in contact with infected persons, and can aid in quantitative analysis of the transmission paths, processes, and trends of the infectious diseases, all leading to the development of corresponding effective epidemic control strategies.

**FIGURE 1.**
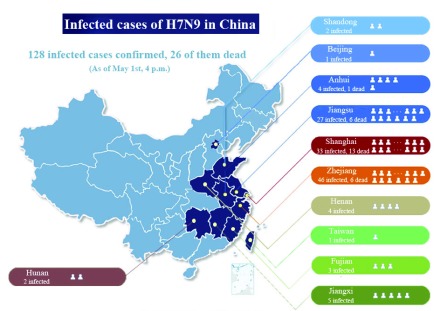
The spatial distribution of H7N9 cumulative cases in the early stage of the outbreak in mainland China in 2013 [10]. In this case, infected cases are recorded with the information of location and time, while the contact information dominating transmission remains unknown.

The biggest obstacle in contact tracking is obtaining data that directly describes contact behaviors. Because contact interactions between individuals are diverse and often subtle, they are difficult to be directly observed and recorded. In other words, it is hard to obtain first-hand high-quality data for contact tracking. When a disease is spreading, the impact of the disease could be observed, instead of the underlying direct interactions between individuals. For example, during the outbreak of H7N9 bird flu, it is difficult to identify who were infected due to contacts with certain infected people. As shown in Fig. 1, only new H7N9 cases and the number of deaths in different time and space can be observed.

Many epidemiology scholars and computer scientists have conducted research on how to accurately capture individuals’ contact behavior data as well as how to indirectly infer the contact network from other data sources. Many methods have been proposed, most of which utilize intelligence data analytics related technologies, such as intelligent sensing, network modeling and analysis, data visualization, multi-source heterogeneous data mining, data-driven reverse engineering, machine learning, and multi-agent simulation, among others. Based on the granularity of contact modeling, the existing methods can be classified into four categories: static individual contact tracking, dynamic individual contact tracing, static group contact tracking, and dynamic group contact tracking. Each of these methods are described and discussed separately in following sections.

## II. Research Status Analysis

### A. Individual Contact Tracking

Individual contact tracking records fine-grained “individual-to-individual” contact information, such as contact time, location, frequency, duration, etc. The most common ways to gather contact information are non-automatic methods, e.g., offline and online questionnaires [11], [12], [24], and automatic methods, e.g., mobile phone, wireless sensors, RFID, and GPS [5], [16], [18], [21].

#### 1) Static Individual Contact Tracking

Offline questionnaire has been used for many years in some counties to trace sexual contacts of sexually transmissible infections (STIs), particularly for HIV [31].

In recent 30 years, HIV has killed more than 30 million people. Currently, there are still 2 million newly infected HIV individuals and half of them will be died every year [32], [33]. To reveal the spread patterns of HIV infection and AIDS in the U.S., Fay et al. analyzed the patterns of same-gender sexual contact among men using the data developed from a national sample survey in 1970 and 1988, respectively. They found that at least 20.3 percent of adult men have had sexual contact with another man in life, and never-married men are more likely to have same-gender sexual contacts [34]. Similarly, Merino *et al.* [35] sampled 294 homosexual men from Colombia as volunteers to answer questionnaires on sexual practices. Analysis of these questionnaires suggests two significant risk factors for HIV-1 infection: 1) having sexual contact with foreign visitors; 2) having more than ten homosexual partners. They suggest that the spread of HIV-1 infections should be monitored at the international level and more attention should be paid to these subgroups with high transmission rates.

In general, most of us tacitly approve that unsafe sexual behavior would be more prevalent among individuals with optimal viral suppression. In the Swiss Cohort Study on April 1, 2000, Wolf *et al.* [36] investigated the unsafe behavior among 4948 HIV-infected individuals by self-reported questionnaire. However, after adjustment for covariate, it reported that unsafe sex is associated with other factors, e.g., gender, age, ethnicity, status of partner, having occasional partners, living alone, etc. In recent years, researchers designed questionnaires to measure the validity and reliability of sexual abstinence and avoidance of high-risk situations. For example, Najarkolaei *et al.* [37] sampled 348 female undergraduate students from Tehran University, Iran, and assessed the validity and reliability of the designed sex, behavioral abstinence, and avoidance of high-risk situation. Zhang *et al.* [38] surveyed 250 HIV-positive persons on their socio-demographic characteristics and sexual behavior, and traced HIV infection status of 431 persons who had heterosexual contact with the HIV carriers. Among these 431 persons, 59 were HIV-positive, i.e., the secondary attack rate was 13.7%. Therefore, they appeal to improve the knowledge about HIV/AIDS, enhance psychological education, and promote the use of condom, so as to suppress the transmission of HIV.

In addition to HIV, offline questionnaire has also been used to trace the contact between individuals to investigate other infectious diseases such as Chlamydia trachomatis infection, Zika, and flu.

To seek the view of patients with Chlamydia trachomatis infection on legislation impinging on their sexual behavior, an investigation was performed on 192 patients at STD clinics in Stockholm, Sweden in 1997. During the past 6 months, men (40%) were more likely to have sexual intercourse with occasional partners than women (21%), and the mean number of men and women was 2.3 and 2.2, respectively [39]. The Zika virus is primarily transmitted by Aedes species mosquitoes. However, by reviewing the travel experience and sexual intercourse of infected individuals in the US, researchers confirmed that there were 14 cases of Zika virus infection were transmitted by sexual contact [40]. For instance, a person in Texas was getting infected with the Zika virus after sexual contact with someone who had acquired the infection while travelling abroad [41]. In 2012, Molinari *et al.* [7] investigated the contact interactions of 1,074 students in a high school through questionnaires. A local campus contact network was established based on information such as the length of contact time and contact frequency. The outbreak process of flu was then simulated based on this established contact network. They found that the classroom is the location with the most campus contact and that class break and lunch break are the times with the most campus contact.

Offline questionnaire is an efficient way to trace private contact interactions such as sexual practice between individuals. However, it needs to find target participants one by one within a specific region, which is time consuming and needs more physical labor. Moreover, data collected by this method is usually time delayed and incomplete. With the purpose of collecting more timely and low-priced data of various kinds of contact behaviors, online questionnaires such as online survey and web-based survey have been extensively applied.

In 1998, a national online survey was constructed in 1690 adolescent males, using computer-assisted self-interviewing (audio-CASI) technology. Comparing with traditional self-administered questionnaire, the prevalence of male-male sex with intravenous drug users estimated by audio-CASI was higher by more than 3% [42]. Influenza-like illness (ILI) outbreaks on a large scale every year in many countries, recording and detecting ILI are important public health problems. Flutracking, a weekly web-based survey of ILI in Australia, has been used to record the past and current influenza immunization status of participants in winter influenza seasons for many years [43]. It only takes the participants less than 15 seconds to complete the survey, including documenting symptoms, influenza vaccination status, and mobility behaviors such as time off work or normal duties. In 2008, the peak week detected by Flutracking was 31 August, which was contemporaneous with that in other influenza surveillance systems [44]. For the first three years being applied, the participants increased from 394 to 982 and 4,872 in 2006, 2007, and 2008, respectively, due to its convenience in completing the survey and its accuracy in detecting the peak week of influenza activity. Flutracking also provides vaccine effectiveness analysis by investigating the status of vaccinated and unvaccinated participants. In 2012, the ILI weekly incidence peaked in mid-July in the unvaccinated group, 1 month earlier than vaccinated group confirmed by national influenza laboratory [45]. In recent years, by cooperating with the health department, organizational email systems, and social media, Flutracing gained over 1000 new participants each year by sending invitations from existing participants. As a result, the number of online participants in Flutracing has exceeded 26,000 in 2016 [43].

Contact information collected through an online questionnaire is more timely and low-priced than offline questionnaire. However, it still cannot record real-time contact information, and, moreover, contact information collected online sometimes inaccurate or even false [46]. Because people on the Internet are usually anonymous, which is incapable to verify the information of their real name, age, place of residence, etc.

#### 2) Dynamic Individual Contact Tracking

Individual contact information obtained through offline or online questionnaires is usually time delayed, incomplete, and inaccurate. With the aim to collect dynamic, complete, and accurate individual contact information, some researchers began to use mobile phone, wireless sensors, RFID, and GPS devices to track individual contact behaviors.

In recent years, the application of mobile phones has become increasingly universal, providing a convenient way to record real time location information [58], [59]. In 2011, Yoneki [16] developed the first contact pattern discovery software, FluPhone, which could automatically detect and record the contact behavior between users by mobile phone. The researchers collected the contact information of 36 users on the Cambridge University campus with this software and established the contact network between different users at different times. Then, they simulated an influenza outbreak on this network using a SEIR model. In view that the large power consumption of GPS and Bluetooth resulted in short standby time of mobile phones, Yoneki and Crowcroft [17] further developed a new contact tracking application, EpiMap, using wearable sensors, which had lower power consumption and longer standby time. EpiMap thoughtfully transmits and stores data by satellite, as many high-risk areas are in developing countries where there often are not enough wireless communication facilities to support contact tracking.

Wearable wireless sensors can record individuals’ contact events such as time, location, and duration continuously and accurately, and gradually becomes a useful tool for collecting high-precision contact data in small areas [20]. It has been applied to discover contact patterns in various kinds of social settings such as hospitals and campuses. For example, MIT Media Lab researchers Nathan Eagle and Alex Pentland proposed the reality mining method as early as 2006. This method suggests the use of wearable wireless sensors to record people’s daily activities [13], [14]. They developed an experimental system to record the activities of several MIT students in a teaching building over time, and then established a small social network describing their contact relationships [15]. Salathé *et al.* [18] collected the contact interactions of 788 students in a high school in the United States for one day using wireless sensors, and they established an individual-based campus contact network. It was found that the campus contact network had high density connectivity and a small-world structure [19]. However, it is costly to trace contact interactions using wearable wireless sensors, especially when the number of individuals being monitored is large. Moreover, people wearing wearable wireless sensors are very conspicuous, participants are unwilling to wear such devices due to privacy concerns especially for patients.

**FIGURE 2.**
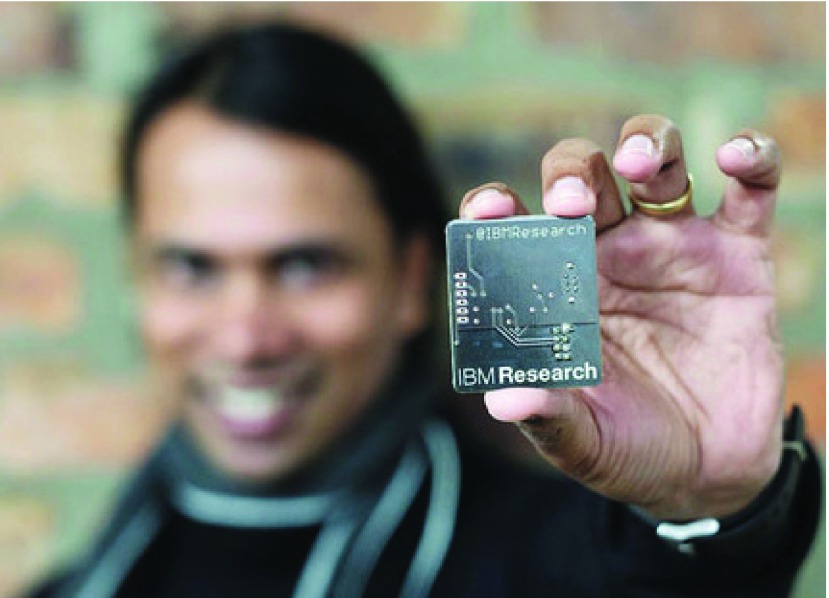
An IBM researcher holds a micro-radio tuberculosis tracker [6]. In October 2016, IBM researchers from Johannesburg, South Africa, released their latest research update: using cheap radio tags to anonymously track the contact transmission paths of tuberculosis. This study is an important step for IBM in helping WHO eliminate tuberculosis.

Radio frequency identification (RFID) is a non-contact automatic identification technology, by which the contact behavior can be recorded when individuals carrying a small non-contact chip getting closer. In 2009, Olguin *et al.* [15] collected 16,000 contacts among 119 people (including medical staff, children in critical condition, and nursing staff) in a children’s hospital in the United States using radiofrequency identification devices (RFID), and established a contact structure for the hospital. Similarly, Yoneki [16] collected students’ contact data from a French primary school using radiofrequency identification devices. More recently, in October 2016, IBM researchers Kurien and Shingirirai from Africa Labs invented a radio tag designed to extend tracker working distance, and implemented it in tracking tuberculosis (Fig. 2) [6]. Each tag contains a tiny sensor, a storage device, and a battery. Radio tags can communicate with each other, allowing individuals’ contact interactions to be recorded when two tags are in close proximity. The contact data collected by the radio tags is presented in a three-dimensional visualization system. Using of the intelligent data analysis method provided by the system, medical staff can view the spatiotemporal distribution of tuberculosis patients in real time, track the transmission paths of tuberculosis, and find high-risk populations. Because of the high cost of tuberculosis vaccines, contact data can also aid in the determination of high-priority vaccinations. However, the traditionally used radiofrequency tracker has a limited transmission and receiving range and only works within a small area.

GPS (Global Positioning System) has the capability of long-distance positioning, which has been widely used for tracing indoor and outdoor mobility behaviors and physical activities [55], [56]. With the aged tendency of population, tracing mobility behavior is critical for measuring, describing, and comparing mobility patterns in older adults. For example, Hirsch *et al.* [47] investigated the mobility patterns using GPS tracing data collected from 95 older adults in Vancouver, Canada, with the goal of understanding neighborhood influences on older adults’ health. They found that participants who were younger tend to drive more frequently and live far from their neighborhoods. GPS devices have also been used for tracing physical activities of adolescents in school and other social settings [48]–[50]. For instance, Rodriguez *et al.* [49] sampled 293 adolescent females in Minneapolis and San Diego, USA, and traced their physical activity and sedentary behaviors by GPS every 60s in different settings. Physical activities were more likely to occur in parks, schools, and places with high population density during weekend, less to occur in places with roads and food outlets. Besides, tracing animals in the sea or on the land using GPS devices can obtain detailed spatiotemporal data regarding the movement patterns. For instance, Dujon *et al.* [51] traced a green turtle travelling more than 300002km across the Indian Ocean and obtained more than 450,2000 locations. Moreover, by tracing the whole-body motion dynamics of a cheetah using GPS devices, Patel *et al.* [52] illuminated the factors that influence performance in legged animals.

Although detailed individual contact information can be collected through non-automatic methods, e.g., offline and online questionnaire, and automatic methods, e.g., mobile phone, wearable wireless sensors, RFID, and GPS devices. These methods are mostly limited to small-scale population experiments due to high cost and short range collection. They have not been applied to large areas or large-scale contact behavior studies.

### B. Group Contact Tracking

Group contact tracing captures contact interactions of human beings with similar characteristics (e.g., age, occupation, hobbies) in different social settings from the macroscopic level. Static group contact behavior can be traced by large-scale questionnaire and simulated by multi-agent models. Dynamic group contact behavior can be inferred by data mining method like tensor deconvolution.

#### 1) Static Group Contact Tracking

In recent years, a composite group model that can characterize population heterogeneity and model epidemic spreading dynamics, overcoming the difficulty of obtaining fine-grained individuals’ data has attracted much attention. Such models not only simulate the transmission process, but also depict the contact structure of a larger population.

**FIGURE 3.**
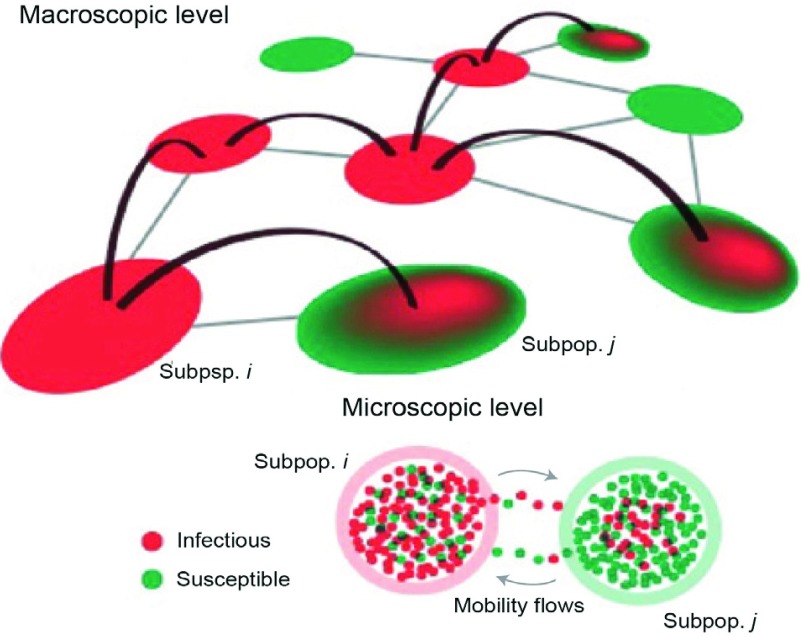
Reaction diffusion model of composite groups [23]. The diffusion process is illustrated from a macroscopic perspective, i.e., the transmission among different meta-populations, whereas the reaction process is illustrated from a microscopic perspective, i.e., the individual infection within a meta-population.

The composite group model divides the population into several meta-populations by age or spatial location, so that individuals within a meta-population have similar biological characteristics (such as susceptibility, infectivity, latent period, and recovery period). Then, the process of epidemic transmission can be modeled using group contact interactions among meta-populations instead of individuals’ contact interactions [23]. Based on this model, the infection and spread of epidemics can be described as a reaction-diffusion process. “Reaction” characterizes the process of individual infection within a meta-population. “Diffusion” characterizes the transfer process of epidemic diseases between different meta-populations through the group contact structure (Fig. 3). In addition, there is a practical significance in establishing contact networks for composite groups because control strategies for epidemic diseases are usually oriented towards composite populations, for example, vaccination groups are usually sectioned by age when planning vaccine allocation strategies.

**FIGURE 4.**
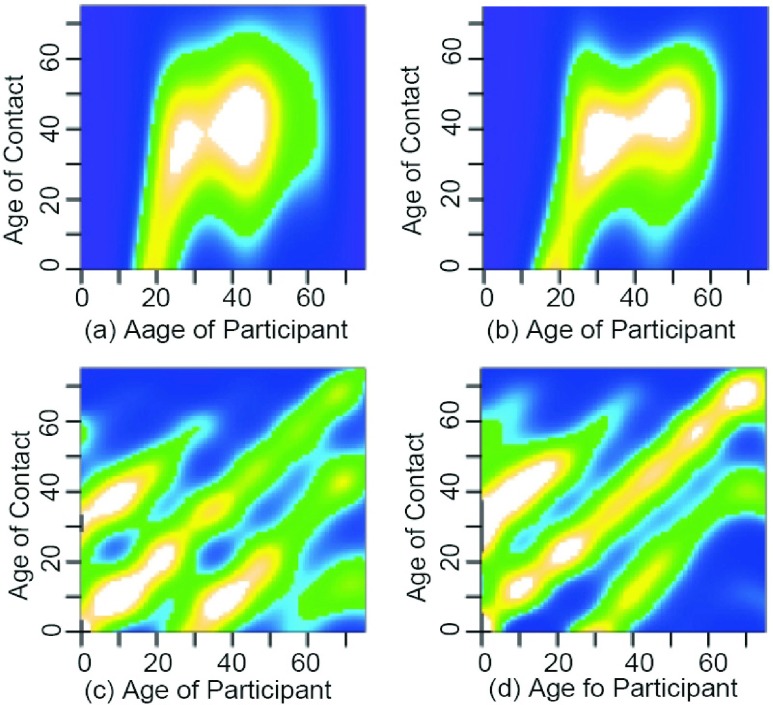
The contact matrix of composite group [24]. The interaction probabilities between different age groups are represented by (a) and (b) for the scene of household in Great Britain and Germany, respectively, and, (c) and (d) for the scene of workplace in Great Britain and Germany, respectively. White, green, and blue indicate high, intermediate, and low contact rates, respectively.

The composite group contact network was first established using questionnaires. In 2008, Mossong *et al.* [24] conducted the Polymod research project in Europe, in which they organized a wide-range survey on contact behaviors, involving 7,290 participants from eight European countries. A total of 97,904 contact records were collected. They found that contact interactions have significant spatial heterogeneity, with most individual contacts occurring at home (23%), work (21%), school (14%), places of entertainment (16%), and while using transportation (3%). Further, contact structures under different scenarios have obvious differences. There are some age-related contact patterns: in many scenarios (such as in schools), individuals are more likely to contact people of similar age; most of the contact between children and their parents occurs at home, while most contacts for adults occur in workplaces. The researchers thus divided the population into several meta-populations, establishing a composite group model based on age. Interaction probabilities between different age groups were estimated according to questionnaire data (Fig. 4), and a contact network based on composite groups was established.

The simulation method based on multi-agent models is also applied to the establishment of contact networks. This generally involves combining the questionnaire survey with population census data to establish the contact structure of composite groups. Iozzi *et al.* [9] modeled a virtual society with the characteristics of Italian society based on questionnaire data from 55,773 people. Human daily migration behaviors were simulated by a virtual community, and a contact matrix of the composite group was obtained. Based on this matrix, the outbreak process of Italian B19 (human parvovirus) was successfully simulated. Similarly, Eubank *et al.* [26] simulated the movement of individuals within a city by large-scale agent system, and then modeled a group contact structure based on their simulation. The data they used included population census data, land usage, population migration, and other daily behavior data.

Constructing contact matrix for meta-population requires large-scale even nationwide questionnaire survey, which is quite costly and time delayed. Multi-agent method simulates human mobility behaviors in the virtual world based on the contact matrix constructed using the data from the real world of the past [22]. It doesn’t consider the changes of existing contact patterns caused by human self-awareness and epidemic-control strategies in the future.

#### 2) Dynamic Group Contact Tracking

Most of the above studies focus on static properties of contact behaviors, such as the contact object (who is contacting), scene (where this contact happens), frequency, and duration. In other words, the aforementioned studies assume that the contact patterns of the individual remain stationary. However, contact interactions usually change with time, and show different temporal and spatial patterns. For example, contact interactions can change periodically with the weather and season, vary significantly between workdays, weekends, and holidays, and may be adjusted in response to the threat of an epidemic disease and during the outbreak by reducing travel or wearing face masks [26], [27]. Additionally, government-imposed epidemic-control strategies can significantly change individuals’ contact patterns [17], [23], [24], [30]. For example, during the outbreak of H1N1 flu in Hong Kong in 2009, interventions, such as flight reductions, school closures, and vaccination efforts, significantly altered individuals’ contact interactions [27]–[29].

Dynamic contacts between individuals are more difficult to be observed and recorded than static contacts because of the limitations of existing contact tracing methods. Offline and online questionnaires are incapable of recording real-time contact information, and usually time delayed to receive feedback from participants. Automatic contact tracing methods such as mobile phone, wearable wireless sensor, RFID, and GPS devices can collect continuous mobility information [53], [60]. However, all these methods are limited to monitoring mobility behaviors for small-scale population, due to the large consumption of power, short range of positioning, high cost of money, etc. Besides, most people cannot be expected to agree to have their dynamic contact interactions monitored in real time because of privacy issue. For example, wearing a tracker can also be equated to declaring one’s self an infectious disease patient. Tuberculosis patients in African countries are branded with social prejudice, making wearing an identifier a sensitive issue [6]. In light of these obstacles, a new path that does not “directly” capture and record individuals’ dynamic contact behaviors, but “indirectly” infers the dynamic contact model of a large-scale population from other readily available data sources must be found.

**FIGURE 5.**
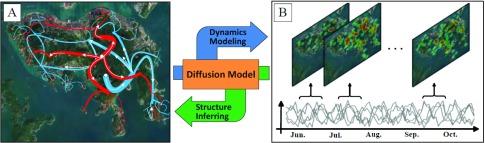
Traditional method and inverse engineering analysis method for modeling, analyzing, and inferring of infectious disease. (A) Traditional method: modeling and analysis of infectious disease by given contact network. (B) Inverse engineering: inferring dynamic social contact patterns using temporally observed incidences.

Infectious disease surveillance, like that depicted in Fig. 1, expands everyday with the vast applications of information technology in the medical field. Surveillance data record spatiotemporal information related to the spread of infectious diseases, which is the result of the spread model acting on the real contact network, as shown in Fig. 5(A). Such surveillance data can be regarded as an external manifestation of the implicit contact network, suggesting that the dynamic contact network could be “inversely” inferred from infectious disease surveillance data, as shown in Fig. 5(B). Essentially, this is a complex inverse engineering problem: using the observed dynamics phenomenon to determine the dynamic structure that leads to the phenomenon. In other words, determining time-dependent contact interactions using the time-dependent spread trend of infectious diseases.

Based on the idea of inverse engineering, Yang *et al.* [25] proposed a novel modeling and inference method for constructing a dynamic contact network based on tensor model. They described the spatiotemporal patterns of composite group contacts as a tensor, modeled the inference of the dynamic contact network as low-rank tensor reconstruction problem, and proposed a tensor deconvolution based inference method by fusing compression perception, sparse tensor decomposition and epidemic propagation models. This method makes it possible to determine the dynamic contact network of the large-scale composite group from population census data and surveillance data of many epidemic diseases.

Using this method, composite group dynamic contact networks for Hong Kong and Taiwan were established using population census data and surveillance data of a variety of infectious diseases (such as H1N1, influenza, measles, mumps, etc.) for these two areas. The temporal and spatial evolution patterns of individuals’ dynamic contact interactions were analyzed. Based on the established dynamic contact network, they further studied the spread law, and prevention and control strategies of H1N1 epidemic disease. They arrived at two important conclusions: (1) in the H1N1 outbreak in Hong Kong in 2009, if the beginning of the new semester was delayed two to six weeks, the total number of infections would have been reduced by 10% to 25%; (2) the best strategy for prevention and control of H1N1 spread is vaccination of school-age children in the first few days of the new semester.

## III. Discussions and Directions

Contact tracking based on intelligent information processing technology represents an active prevention and control strategy for infectious diseases. Its main functions are to achieve early detection and timely intervention of infectious diseases. Research on contact tracking methods not only expands the options for preventing and controlling infectious diseases, but also further improves people’s understanding of their own contact behaviors.

Contact tracking has become an increasingly mature data-driven technology for disease prevention and control, evolving from individual tracking to group tracking. Individual tracking attempts to capture more detailed contact interactions for accurate locating of infected patients and high-risk susceptible populations. Traditional offline questionnaire is a practical method for tracing private contact interactions between individuals such as sexual practice. However, it is quite costly and time delayed to find target participants and receive feedback from them. Comparatively speaking, online questionnaire serves a low-priced way to collect feedback from participants timely. However, it cannot record the time exactly when contact occurs. Meanwhile, offline and online questionnaires sometimes provide inaccurate information of human mobility. For example, Klous *et al.* [46] surveyed 870 participants in a rural in the Netherlands using questionnaire and GPS logger, respectively. Investigations on walking, biking, and motorized transport duration showed that time spent in walking and biking based on questionnaire was strongly overestimated.

The use of automatic contact tracing methods enabled researchers to obtain continuous and accurate individual contact information, e.g., time, location, duration, etc. Mobile phone and wireless sensors were widely used to trace mobility behaviors of students in campus and patients in hospital. Then, small-scale contact network within the tracing regions can be constructed and the diffusion process of infectious disease such as influenza can be simulated and analyzed in detail. However, the use of mobile phone is limited to tracing short-term contact behavior because of large power consumption of GPS and Bluetooth. Wearable wireless sensors can only be applied to small-scale population due to its high cost and privacy concerns. RFID devices are convenient carrying which solves privacy concerns very well, but it only can be used for short range collection. GPS device has the advantage of long-distance positioning. However, it is costly to capture indoor mobility behaviors due to the requirement of communication stations [57]. All these automatic contact tracing methods have not been used for studies of large-scale individual contact so far.

Group tracking replaces individual contacts with the contact probability of meta-populations, which, to some extent, overcomes the obstacles of individual tracking. Using a contact matrix of meta-population, contact patterns regarding people with similar features can be depicted from the macroscopic level. However, the contact matrix is usually constructed using the data collected from a nationwide questionnaire, which is static and can only represent the contact patterns of the past. To explore dynamic contact patterns of meta-population, a data-driven AI (artificial intelligence) method was adopted, i.e., tensor deconvolution [25]. Based on this method a dynamic evolutionary model of the group contact was constructed and dynamic contact patterns were inferred inversely through insights into the time-dependent nature of the infectious disease surveillance data. Nevertheless, it should be noted that although it can characterize a wider range of dynamic contact behaviors, it cannot be used to accurately locate unique contact events because of the coarse granularity of the captured contact behaviors.

Exploring social contact patterns for epidemic prevention and control is an every promising research direction, and some potential future development directions are illustrated as follows.

### A. Multi-View Contact Tracing

Data obtained from different views can give expressions to different patterns of mobility behaviors. For example, offline and online questionnaire can accurately record contact events occurred in places that individuals frequently visited [53]. GPS devices can record indoor and outdoor contact events happened occasionally [54]. Heterogeneous contact network constructed by various kinds of information can provide a new way for analyzing and simulating the spread of epidemics. Therefore, tracing mobility behaviors and analyzing contact patterns from multi-views to get new insight into what heterogeneous contact patterns like will be a new direction in the future.

### B. Multi-Scale Contact Tracing

Existing studies focus on either individual level or group level contact tracing, presenting independent contact patterns from microscopic and macroscopic scales, respectively. However, group contact patterns are formed by collaborative behaviors of individual mobility, while individual mobility behaviors can be influenced by others in the same group. Revealing hidden interactions between individual contact and group contact will be helpful to identify influential individuals as sentries for disease monitoring. Therefore, discovering hidden interactions from multi-scale contact patterns that tunneling individual contact and group contact will be a new opportunity for early epidemic detection.

### C. AI-Based Contact Tracing

Dynamic mobility behaviors lead to complex contact patterns, which are usually hidden and cannot be directly traced by non-automatic or automatic methods. A better way to infer dynamic contact patterns is adopting AI-based methods using heterogeneous real-world data. Existing studies such as tensor deconvolution consider the combination of contact probabilities within real-world social settings like school, home, and workplace as linear [25]. However, hidden dynamic contact patterns within these social settings could be more complicated than linear models can characterize. Therefore, exploring advanced AI-based contact tracing methods, e.g., multi-view learning [61]–[64], deep learning [65], broad learning [66], etc., will be the next generation technology for epidemic prevention and control.

## IV. Conclusions

In this paper, we introduced current studies on contact tracing and its applications in epidemic prevention and control. This paper covered 2 research directions, i.e., individual contact and group contact, which were introduced from both static and dynamic aspects. Non-automatic tracing methods like offline and online questionnaires record static individual contact information, while automatic tracing methods like mobile phone, wearable wireless sensor, RFID, and GPS devices collect dynamic contact events. Static group contact patterns can be depicted by a coarse granularity contact matrix constructed by large-scale questionnaire data, dynamic contact patterns, however, can only be inversely inferred using data-driven AI technologies. Both individual and group contact tracing are promising research directions and filled with challenges, especially for dynamic contact tracing. Collecting contact data from multi-views and analyzing contact patterns from multi-scale mobility interactions will be new directions in the future. Moreover, exploring advanced AI-based contact tracing methods using heterogeneous and multi-source data will provide new opportunities for epidemic prevention and control.
